# Supplementary material for: Experiences of Migrant People Living with HIV in a Multidisciplinary HIV Care Setting with Rapid B/F/TAF Initiation and Cost-Covered Treatment: The ‘ASAP’ Study
Source: J Pers Med. 2022 Sep 13;12(9):1497. doi: 10.3390/jpm12091497 (PMC9503330; doi:10.3390/jpm12091497)
Supplement: Supplementary file 1 [file jpm-12-01497-s001.zip › jpm-1900609-supplementary.pdf]

## Supplementary Materials

### Interview Week 1

1. What is your experience of beginning HIV treatment?
2. Currently, how satisfied are you with your:
    a. HIV treatment ?
    b. HIV care ?
3. How can we improve the HIV care you are receiving ?
4. What worries do you have about your:
    a. HIV treatment ?
    b. HIV care?
5. What benefits do you expect from your:
    a. HIV treatment ?
    b. HIV care?

### Interview Week 24

1. Tell me about your first visits at the clinic; and about your first weeks on your current HIV treatment.

Prompt questions:

How did you feel?

What barriers and difficulties did you face when came for the first times at the clinic? When taking the treatment?

What made it easier for you to come at the clinic? To take the treatment?

If the participant recently immigrated to Canada:

What procedures did you follow relative to immigration?

How do you think your HIV status may impact your integration process in the future?

2. How did your general situation evolve since you began receiving care at the clinic? Since you initiated your HIV treatment?

Prompt questions:

At this moment, to which extent do you feel similar to how you felt at the time? To which extent do you feel different?

What is different or similar in terms of… ?

… social connexions, networks, status

… lifestyle, organization, daily activities

… quality of life, wellbeing, personal fulfilment

… access to social services, to health care

… your emotional/physical/social health, ability to function

3. What service or staff at the clinic contributed to positive or negative changes in your situation? What service or staff at the clinic made it easier or more difficult for your to take the treatment? How?

4. What do you think about the care and treatment that you are taking?

What are the negative aspects? What are the positive aspects? What could be improved?

If the participant recently immigrated to Canada:

What are the positive aspects of the immigration process? What are the negative, or difficult aspects of the immigration process?

How did this process impact the care that you receive and your uptake of the treatment?

What kind of services could be provided at your HIV clinic, to facilitate this process?

**Interview Week 48**

1. To which extent are you responsible of managing your care and treatment? To which extent are your care providers responsible?

Prompts:

How do you define your responsibilities? How do you define theirs?

What actions can you take to improve your experience of care and of the treatment? What actions can they take?

2. To which extent do you feel you can talk to your care providers about the positive aspects of the care that you receive and of the treatment? About their negative aspects?

Prompts:

To which extent can you ask them for assistance when you face a problem?

To which extent can you manage problems yourself?

What solutions did you find to problems with care and treatment? What solutions were provided by care providers?

3. To which extent have you maintained a health condition and lifestyle similar to when you initiated care and treatment? To which extent are your health condition and lifestyle different?

Prompts:

How did your health condition and lifestyle change? How did your treatment impact them?

4. How could the services that you receive at the clinic be improved? What should change in the clinic? What should be kept?
